# “Pseudo-Beijing”: Evidence for Convergent Evolution in the Direct Repeat Region of *Mycobacterium tuberculosis*

**DOI:** 10.1371/journal.pone.0024737

**Published:** 2011-09-13

**Authors:** Lukas Fenner, Bijaya Malla, Béatrice Ninet, Olivier Dubuis, David Stucki, Sonia Borrell, Thembela Huna, Thomas Bodmer, Matthias Egger, Sebastien Gagneux

**Affiliations:** 1 Institute of Social and Preventive Medicine, University of Bern, Bern, Switzerland; 2 Department of Medical Parasitology and Infection Biology, Swiss Tropical and Public Health Institute, Basel, Switzerland; 3 University of Basel, Basel, Switzerland; 4 Laboratory of Bacteriology, University Hospital of Geneva, Geneva, Switzerland; 5 Viollier AG, Basel, Switzerland; 6 MRC National Institute for Medical Research, London, United Kingdom; 7 Mycobacteriology Unit, Institute for Infectious Diseases, University of Bern, Bern, Switzerland; Universita di Sassari, Italy

## Abstract

**Background:**

*Mycobacterium tuberculosis* has a global population structure consisting of six main phylogenetic lineages associated with specific geographic regions and human populations. One particular *M. tuberculosis* genotype known as “Beijing” has repeatedly been associated with drug resistance and has been emerging in some parts of the world. “Beijing” strains are traditionally defined based on a characteristic spoligotyping pattern. We used three alternative genotyping techniques to revisit the phylogenetic classification of *M. tuberculosis* complex (MTBC) strains exhibiting the typical “Beijing” spoligotyping pattern.

**Methods and Findings:**

MTBC strains were obtained from an ongoing molecular epidemiological study in Switzerland and Nepal. MTBC genotyping was performed based on SNPs, genomic deletions, and 24-loci MIRU-VNTR. We identified three MTBC strains from patients originating from Tibet, Portugal and Nepal which exhibited a spoligotyping patterns identical to the classical Beijing signature. However, based on three alternative molecular markers, these strains were assigned to Lineage 3 (also known as Delhi/CAS) rather than to Lineage 2 (also known as East-Asian lineage). Sequencing of the RD207 in one of these strains showed that the deletion responsible for this “Pseudo-Beijing” spoligotype was about 1,000 base pairs smaller than the usual deletion of RD207 in classical “Beijing” strains, which is consistent with an evolutionarily independent deletion event in the direct repeat (DR) region of MTBC.

**Conclusions:**

We provide an example of convergent evolution in the DR locus of MTBC, and highlight the limitation of using spoligotypes for strain classification. Our results indicate that a proportion of “Beijing” strains may have been misclassified in the past. Markers that are more phylogenetically robust should be used when exploring strain-specific differences in experimental or clinical phenotypes.

## Introduction

*Mycobacterium tuberculosis* complex (MTBC) adapted to humans consist of six main phylogeographical lineages [1]. There is increasing evidence that strain diversity in MTBC plays a role in the outcome of infection and disease in tuberculosis (TB) [2], [3]. One particular MTBC genotype known as “Beijing” has repeatedly been associated with drug resistance [4] and increased virulence in animal models [2]. This genotype was first described in 1995 [5], and has traditionally been defined based on a characteristic spoligotyping pattern [6]. More recently, phylogenetic analyses showed that the Beijing strain family belongs to Lineage 2 (known as East Asian lineage), which is one of the six main human-adapted lineages of MTBC [7], [8]. Beijing strains are most often isolated in East- and Southeast Asia, in countries of the former Soviet Union, and have recently been emerging in South Africa [1], [6], [9], [10].

Spoligotyping is based on the Clustered Regulatory Short Palindromic Repeats (CRISPR) region known as the Direct Repeat (DR) locus in MTBC. This region is characterized by series of direct repeats interspersed by short unique regions called “spacers” [11]. The characteristic spoligotyping pattern of Beijing strains reflects the loss of the first 34 spacers of a total of 43 used in standard spoligotyping [5], [10]. Repetitive DNA sequences like the DR locus exhibit a high rate of change, and convergent evolution can lead to identical genetic character states in phylogenetically unrelated strains; a phenomenon referred to as homoplasy [8].

We recently identified novel SNP markers that define the main phylogenetic lineages [8], [12]. In contrast to spoligotyping, SNPs in MTBC exhibit almost no homoplasy [8]. Here we used these SNPs, combined with genomic deletion and MIRU-VNTR analyses to revisit the phylogenetic classification of MTBC strains exhibiting the classical “Beijing” spoligotyping pattern.

## Methods

MTBC isolates were obtained during an ongoing population-based study on the molecular epidemiology of TB in Switzerland, and from an ongoing hospital-based study in Kathmandu, Nepal.

Mycobacterial isolates were cultured and DNA extracted according to standard laboratory procedures. Spoligotyping was performed as previously described and compared to data published in SpolDB4 [13], [14]. 24-loci MIRU-VNTR was performed as previously described [11], and the data analyzed using the MIRU-VNTRplus online tool (http://www.miru-vntrplus.org). Determination of the main phylogenetic MTBC lineages was performed by TaqMan real-time PCR (Taqman, Applied Biosystems, USA) using primers (Sigma-Aldrich, Buchs, Switzerland), Taqman Universal MasterMix II and Taqman minor groove binder probes (Table 1) targeting lineage-specific SNPs reported previously [8], [12]. Region of difference (RD) deletion PCRs were performed for RD105, RD207 and RD750 [15]. PCR products of RD207 were directly sequenced. All genotyping experiments of the three “Pseudo-Beijing” isolates described here were repeated at least twice by two independent investigators.

**Table 1:** Sequence information of probes and primers used in this study to detect main phylogenetic lineages of *Mycobacterium tuberculosis* complex isolates by single nucleotide polymorphisms genotyping.

Lineage	Alternative name	SNP name*	Primer sequences	Probe sequences
2	East Asian Lineage	Rv2952_0526n	F: 5′-CCTTCGATGTTGTGCTCAATGT-3′	Wild type probe:FAM: 5′-CCCAGGA**G**GGTAC-3′
			R: 5′-CATGCGGCGATCTCATTGT-3′	Lineage-specific probe:VIC: 5′-CCCAGGA**A**GGTACT-3′
				
3	Delhi/CAS	Rv3804c_0012s	F: 5′-GCATGGATGCGTTGAGATGA-3′	Lineage-specific probe:FAM: 5′-AAGAATGCAGCTTGT**C**GA-3′
			R: 5′-CGAGTCGACGCGACATACC-3′	Wild-type probe:VIC: 5′-AAGAATGCAGCTTGT**T**GA-3′

*as reported in Ref. [8].

F: forward; R: reverse; SNP, single nucleotide polymorphisms.

Probes are minor groove binder probes.

The Swiss study was approved by the ethics committee of the Canton of Berne, Switzerland. Written informed consent was obtained from the patient by the treating physicians. In some cases informed consent could not be obtained because the patient could not be located or was known to have died. For these cases we obtained permission from the Federal expert commission on confidentiality in medical research (based at the Federal Office of Public Health, Bern, Switzerland) to use the data provided by the treating physician based on clinical notes. The study in Nepal was approved by the Nepal Health Research Council (NHRC), Kathmandu, Nepal, and the ethics committee of the Canton of Basel, Switzerland. Written informed consent was obtained for all Nepalese patients.

## Results

Among the isolates recovered in Switzerland, we identified a total of 52 that exhibited the characteristic “Beijing” spoligotype. SNP-genotyping confirmed that 50 of these (96.2%) belonged to Lineage 2. Two isolates (3.8%) belonged to Lineage 3 (also known as Delhi/CAS). Similarly, among 55 Nepalese isolates with a Beijing spoligotype, 54 (98.2%) were confirmed as belonging to Lineage 2, while one isolate (1.8%) belonged to Lineage 3 (Table 2). The three Lineage 3 isolates were epidemiologically unrelated and were isolated from HIV-negative patients. The two strains from Switzerland were isolated in patients living in Switzerland but originating from Portugal and Tibet, and the strain from Nepal was isolated in a patient born and living in Nepal. All but one strains were pan-susceptible (one isolate with a *katG* S315T mutation). We found no evidence for a mixed-strain infection or clonal heterogeneity [16] based on the 24-loci MIRU-VNTR pattern.

**Table 2:** Spoligotyping, single nucleotide polymorphism (SNP) and region of difference (RD) PCR results from the three *Mycobacterium tuberculosis* isolates belonging to Lineage 3.

Specimen no.	Spoligotyping pattern (spacers 1–43)	SIT^1^	Clade	SNP for Lineage 2	SNP for Lineage 3	Deletion of RD 105	Deletion of RD 750
1395	□□□□□□□□□□□□□□□□□□□□□□□□□□□□□□□□□□▪▪▪▪▪▪▪▪▪	1	Beijing	−	+	−	+
1476	□□□□□□□□□□□□□□□□□□□□□□□□□□□□□□□□□□▪▪▪▪▪▪▪▪▪	1	Beijing	−	+	−	+
2503	□□□□□□□□□□□□□□□□□□□□□□□□□□□□□□□□□□▪▪▪▪▪▪▪▪▪	1	Beijing	−	+	−	+

1 Spoligotype International Type (SIT) according to the definition in SpolDB4 database using SITVIT2 (http://www.pasteur-guadeloupe.fr:8081/SITVITDemo/index.jsp).

+, present; −, absent; RD, region of difference; SNP, single nucleotide polymorphism.

**Figure 1:**
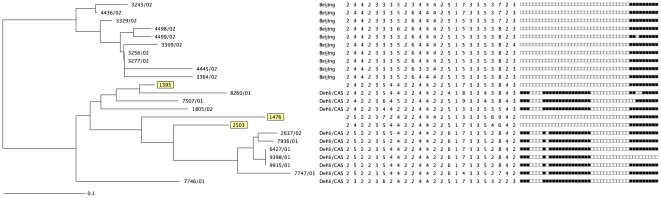
Neighbor-joining dendogram based on the copy numbers of 24 MIRU-VNTR loci using the web-based MIRU-VNTR*plus* tool (http://mvpl.mlvaplus.net). The *Mycobacterium tuberculosis* isolates reported here are highlighted in boxes, reference strains are from the MIRU-VNTR*plus* database. The corresponding spoligotyping patterns are shown as a reference but were not included in the construction of the dendogram.

To confirm our SNP data, we subjected these three isolates to further molecular investigations. Lineage 2 strains, including all Beijing strains, are deleted in RD105 [7], [10], whereas Lineage 3 strains have a deletion in RD750 [7]. We found that the three strains of interest had RD105 intact and a deletion in RD750 (Table 1). In addition, the MIRU-VNTR profiles of these isolates clustered with the Lineage 3 strains (Delhi/CAS) rather than with the Beijing strains (Figure 1). The further investigations thus confirmed that these three strains belonged to Lineage 3 rather than Lineage 2.

**Figure 2:**
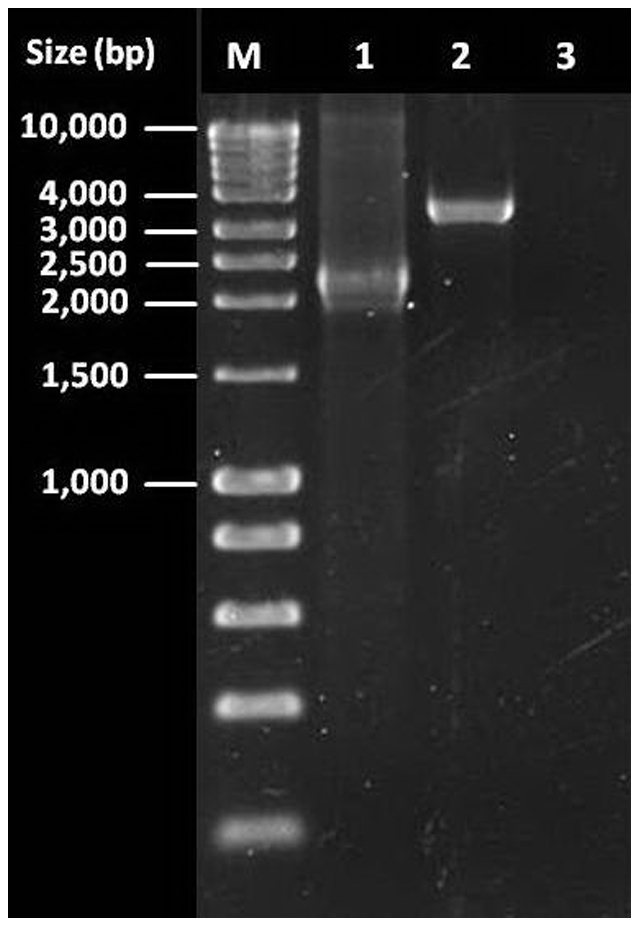
Results of Region of Difference (RD) 207 polymerase chain reaction. M, molecular weight marker; 1, “True” Beijing isolate; 2, “Pseudo-Beijing” isolate no. 1395; 3, Negative control.

We explored the molecular mechanism by which these Lineage 3 isolates acquired their “Pseudo-Beijing” spoligotyping patterns. “True” Beijing strains harbor a 7,399 base pair (bp) deletion in RD207, which is responsible for the missing spacers 1–34 [10]. We thus hypothesized that the strains of interest might have acquired a similar but distinct deletion linked to an independent mutational event. We amplified RD207 [15] but failed to obtain a product in two of the three strains. Strain 1395 for which our PCR was successful yielded a PCR product that was about 1,000 bp larger than the corresponding product seen in true Beijing strains (Figure 2), indicating that the deletion responsible of the “Pseudo-Beijing” spoligotype in 1395 was about 1000 bp smaller than the classical deletion of RD207. Direct sequencing of the PCR product showed that the 3′-deletion boundary was 1,093 bp upstream (GenBank accession no. JF789456) of the deletion end point for RD207 published previously [15]. These results again indicate that the deletion in strain 1395 is distinct from the standard RD207 deletion. We were unable to determine the exact deletion starting point of this new deletion due to the repetitive nature of the DR region.

## Discussion

In this report we present three cases with MTBC isolates harboring spoligotype patterns identical to the Beijing signature, which were assigned to Lineage 3 (also known as Delhi/CAS) rather than to Lineage 2 (also known as East-Asian) based on three alternative molecular markers. We also provide evidence that these strains acquired independent deletion(s) in the DR locus of MTBC.

The fact that phylogenetically unrelated MTBC strains can harbor identical or very similar spoligotyping patterns has been observed before [17], [18]. The DR locus of MTBC is highly variable, and convergent evolution can lead to homoplasy in spoligotyping patterns [8]. Even though strain classification based on spoligotyping will assign MTBC strains to the correct phylogenetic lineages in about 90% of the cases, some strains cannot be classified at all [19], and others will be misclassified as shown here.

Misclassification of “Beijing” strains is particularly relevant given that this strain family has received increased attention over the last few years [6]. In addition to their association with clinical drug resistance and hyper-virulence in animal models [2], [4], Beijing strains have been emerging in Cape Town, South Africa [9], [20], and the Canary islands [21].

The data presented here suggest that a small fraction of strains traditionally referred to as “Beijing” strains might belong to another phylogenetic lineage. We stress that the prevalence of this phenomenon observed in our study of isolates from Switzerland and Nepal will not reflect the global *M. tuberculosis* genetic diversity. Of note, the three “Pseudo-Beijing” strains belonging to Lineage 3 were isolated from patients originating from three different countries. Given the mutational dynamics within the DR locus, and the fact that strains harboring the classical Beijing spoligotyping pattern are rarely verified using independent molecular markers, it is possible that other so-called “Pseudo-Beijing” strains might turn out to belong to yet a different MTBC lineage. Further studies are needed to better define the global molecular epidemiology of these strains, including clinical phenotypes.

In conclusion, our case report illustrates an example of convergent evolution in the DR locus of MTBC, and highlights the limitation of using spoligotypes for genotypic classification. Markers that are more phylogenetically robust should be used when looking for associations between particular MTBC genotypes and experimental or epidemiological variables [2]. Future work should define the global prevalence and phenotypic characteristics of the “Pseudo-Beijing” strains.
